# In Vitro Assessment of the Genotoxic Hazard of Novel Hydroxamic Acid- and Benzamide-Type Histone Deacetylase Inhibitors (HDACi)

**DOI:** 10.3390/ijms21134747

**Published:** 2020-07-03

**Authors:** Annabelle Friedrich, Ann-Sophie Assmann, Lena Schumacher, Jana v. Stuijvenberg, Matthias U. Kassack, Wolfgang A. Schulz, Wynand P. Roos, Finn K. Hansen, Marc Pflieger, Thomas Kurz, Gerhard Fritz

**Affiliations:** 1 Institute of Toxicology, Medical Faculty, Heinrich Heine University Duesseldorf, Moorenstrasse 5, D-40225 Düsseldorf, Germany; Annabelle.Friedrich@uni-duesseldorf.de (A.F.); Ann-Sophie.Assmann@uni-duesseldorf.de (A.-S.A.); Lena.Schumacher@uni-duesseldorf.de (L.S.); javan100@uni-duesseldorf.de (J.v.S.); 2 Institute of Pharmaceutical and Medicinal Chemistry, Heinrich Heine University Duesseldorf, Universitätsstraße 1, D-40225 Düsseldorf, Germany; matthias.kassack@uni-duesseldorf.de (M.U.K.); pflieger@hhu.de (M.P.); thomas.kurz@uni-duesseldorf.de (T.K.); 3 Department of Urology, Medical Faculty, Heinrich Heine University Duesseldorf, Moorenstrasse 5, D-40225 Düsseldorf, Germany; Wolfgang.Schulz@uni-duesseldorf.de; 4 Institute of Toxicology, University Medical Center, Johannes Gutenberg University Mainz, Obere Zahlbacher Str. 67, D-55131 Mainz, Germany; rooswy00@uni-mainz.de; 5 Institute for Drug Discovery, Medical Faculty, Leipzig University, Brüderstraße 34, D-04103 Leipzig, Germany; finn.hansen@uni-leipzig.de

**Keywords:** HDAC inhibitors, normal tissue toxicity, genotoxic hazard, genetic instability, DNA strand breaks, DNA damage response

## Abstract

Histone deacetylase inhibitors (HDACi) are already approved for the therapy of leukemias. Since they are also emerging candidate compounds for the treatment of non-malignant diseases, HDACi with a wide therapeutic window and low hazard potential are desirable. Here, we investigated a panel of 12 novel hydroxamic acid- and benzamide-type HDACi employing non-malignant V79 hamster cells as toxicology guideline-conform in vitro model. HDACi causing a ≥10-fold preferential cytotoxicity in malignant neuroblastoma over non-malignant V79 cells were selected for further genotoxic hazard analysis, including vorinostat and entinostat for control. All HDACi selected, (i.e., KSK64, TOK77, DDK137 and MPK77) were clastogenic and evoked DNA strand breaks in non-malignant V79 cells as demonstrated by micronucleus and comet assays, histone H2AX foci formation analyses (γH2AX), DNA damage response (DDR) assays as well as employing DNA double-strand break (DSB) repair-defective VC8 hamster cells. Genetic instability induced by hydroxamic acid-type HDACi seems to be independent of bulky DNA adduct formation as concluded from the analysis of nucleotide excision repair (NER) deficient mutants. Summarizing, KSK64 revealed the highest genotoxic hazard and DDR stimulating potential, while TOK77 and MPK77 showed the lowest DNA damaging capacity. Therefore, these compounds are suggested as the most promising novel candidate HDACi for subsequent pre-clinical in vivo studies.

## 1. Introduction

Histone/protein deacetylases (HDACs) are involved in the regulation of multiple cellular functions, including gene expression, cell cycle progression, and tumorigenesis as well as protein activity and protein stability [1,2,3,4,5]. They often become deregulated during cancer development and, among others, confer therapy resistance by affecting cell death pathways and DNA repair [1,6,7]. HDACs are sub-grouped into four different classes that require either zinc (Zn^2+^) (i.e., class I, II, and IV) or nicotinamide adenine dinucleotide (NAD^+^)(class III) for their catalytic activity. Class I HDACs comprise the isoforms HDAC1, 2, 3, and 8, class IIA consists of HDAC 4, 5, 7, and 9, class IIB of HDAC6 and 10, class IV of HDAC11, and class III is represented by the sirtuins (SIRT1-7) [3,8]. Some HDACs are up-regulated in cancers, including leukemia, colorectal, breast, lung, prostate, and pancreatic tumors [1,9]. Others are down-regulated in individual tumor specimens, for instance, of the skin, pancreas, or the colon [1]. Because of the comprehensive involvement of HDACs in the regulation of mechanisms participating in tumor initiation and progression, HDAC inhibitors (HDACi) have been approved by the Food and Drug Administration (FDA) for the therapy of hematological malignancies [10,11]. For example, vorinostat and panobinostat have already been approved for the treatment of cutaneous T-cell lymphoma (CTCL) and multiple myeloma, respectively [12,13,14,15]. In addition, HDACi are considered to be useful for the treatment of various solid tumors [7,16,17,18] and in cancer immunotherapy [19]. The majority of HDACi that are currently used in the clinic are pan-HDACi, as represented by the hydroxamic acid-based compounds vorinostat, panobinostat, and belinostat [20,21]. The benzamides entinostat, mocetinostat, or tacedinaline comprise a chemically different class of clinically relevant HDACi, which preferentially inhibit class I HDACs [22,23]. Of note, HDACi can impair genetic stability by epigenetic mechanisms and by affecting the acetylation of proteins that are involved in the regulation of DNA repair and the DNA damage response (DDR), such as X-ray repair cross-complementing protein 6 (Ku-70), Ataxia telangiectasia mutated kinase (ATM), checkpoint kinases (Chk1,2), the nuclear protein kinase WEE1, the tumor suppressor p53, the DNA mismatch repair protein MSH2, and breast cancer-associated proteins (BRCA) [1,24,25,26,27,28]. By interfering with mechanisms of DDR and DNA repair, including the repair of broken replication forks [29], HDACi increase the anticancer efficacy of various conventional (i.e., genotoxic) anticancer drugs and radiation [25,30,31,32,33,34,35,36,37]. Hence, HDACi are suggested as particular powerful anticancer drugs, especially if used in combination treatment regimen [22,36,38,39].

Apart from their desired anticancer activity, conventional tumor therapeutics also evoke numerous agent-specific adverse effects in normal tissue. Regarding possible long-term adverse effects of HDACi, their potency to harm genetic stability is of utmost concern [40], especially for possible indications in case of widespread non-malignant human diseases, such as cardiovascular [41], neurological [20,42,43,44,45], and immunological disorders [46]. Remarkably, these illnesses are currently emerging as promising novel areas of application of HDACi [47,48,49]. Moreover, considering the potential use of HDACi for the therapy of childhood malignancies, for instance, neuroblastoma and medulloblastoma [4,50], possible long-term side effects of HDACi are of appreciable concern for young patients. In particular, the frequently used and highly potent hydroxamic acid-based pan-HDACi are reported as mutagenic on the basis of results obtained by a Salmonella typhimurium-based mutagenicity test (AMES test) and, moreover, cause chromosomal aberrations according to the results of the micronucleus assay [40]. Of note, belinostat has even been demonstrated to promote genetic instability in vivo in mice at human relevant doses [51]. Molecular mechanisms underlying the presumed genotoxicity of pan-HDACi are unclear. It has been speculated that the metabolism of the hydroxamic acid group, which is important for Zn^2+^ binding, gives rise to electrophilic intermediates (i.e., isocyanates) (Lossen rearrangement), which in turn interact with nucleophilic DNA bases, eventually forming bulky DNA adducts [40,52]. In addition, HDACi can indirectly promote genetic instability by affecting DNA repair and DDR [1,24,25,26,27,28], which has been shown to be advantageous for their application in combined radio-chemotherapy. Importantly, however, in view of the aimed broader application of HDACi in the therapy of different types of diseases, including (non-malignant) cardiovascular and neurological disorders, novel HDACi with a reasonably low cytotoxic and genotoxic hazard potential for normal tissue are desirable to prevent conceivable chronic adverse effects. Having this in mind, we (1) comparatively characterized the cytotoxic and genotoxic potency of a set of newly synthesized derivatives of hydroxamic acid- and benzamide-type HDACi, using vorinostat and entinostat as well-established reference compounds and (2) aimed to unravel molecular mechanism(s) that contribute to the genotoxic effects of HDACi. 

## 2. Results and Discussion

### 2.1. The Therapeutic Window of Novel Hydroxamic Acid- and Benzamide-Type HDACi 

**Figure 1:**
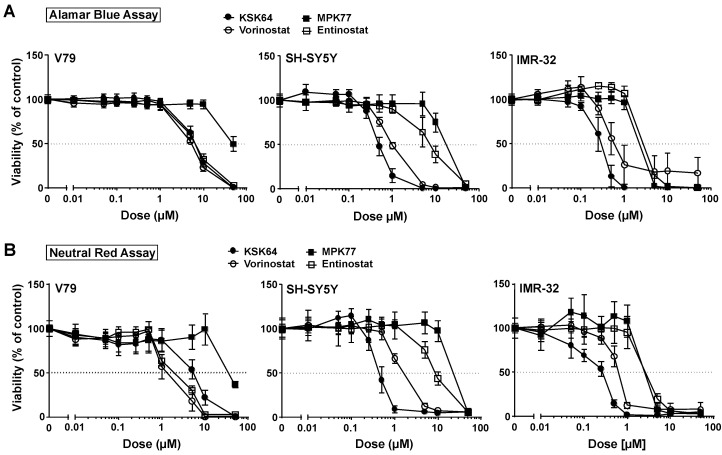
Comparative analysis of the cytotoxicity of novel hydroxamic acid- and benzamide-type histone deacetylase inhibitors (HDACi). Cell viability was analyzed 72 h after administration of representative HDACi candidate compounds (i.e., hydroxamic acid-type HDACi: KSK64, vorinostat; benzamide-type HDACi: MPK77, entinostat) using the Alamar blue and the Neutral red assay. The neuroblastoma cell lines IMR-32 and SY-SY5Y were used as a tumor cell model, whereas V79 lung hamster cells were employed as non-malignant counterpart according to the international OECD (Organisation for Economic Co-operation and Development) guidelines for toxicity testings. (**A**) Cell viability was measured by the Alamar blue assay. Data shown are the mean ± SD from n ≥ 2 independent experiments each performed in quadruplicate (N = 4). (**B**) Cell viability measured by the Neutral red assay. Data shown are the mean ± SD from n ≥ 2 independent experiments each performed in quadruplicate (N = 4).

In the first set of experiments we comparatively analyzed the cytotoxic effects of a panel of novel hydroxamic acid- and benzamide-type HDACi (Supplementary Figure S1), using the established hydroxamic acid-type pan-HDACi vorinostat and the benzamide-type HDAC class I inhibitor entinostat as reference compounds. To monitor cytotoxicity we employed both the Alamar blue assay and the Neutral red assay, which reflect mitochondrial activity and membrane integrity, respectively. V79 hamster lung cells were employed as a non-malignant cell system as suggested by the OECD (Organisation for Economic Co-operation and Development) guidelines for toxicological hazard identifcation. As in vitro tumor cells’ model, human neuroblastoma cell lines (named IMR-32 and SY-SY5Y) were included. A similar degree of HDACi-induced loss of viability was observed by the use of the Alamar blue and the Neutral red assay (Figure 1). The obtained half maximal inhibitory concentations (IC_50_)varied substantially between the chemically different HDACi and the various cell lines (Figure 1 and Supplementary Table S1). Most importantly, the novel hydroxamic acid-type HDAC inhibitory compound KSK64 exhibited a similar toxicity to vorinostat in non-malignant V79 cells while showing higher cytotoxicity in neuroblastoma cells as evidenced by the Alamar blue assay (Figure 1A). In the Neutral red assay, KSK64 provoked even lower cytotoxicity in V79 cells than the approved drug vorinostat (Figure 1B). Overall, these data point to an improved therapeutic window of KSK64 over the clinically approved drug, vorinostat. 

The novel benzamide-type HDACi MPK77 was much less cytotoxic in normal V79 cells than the reference substance entinostat (Figure 1A,B), while showing a similar toxicity to entinostat in IMR-32 cells (Figure 1A,B). Hence, the data again point to an improved therapeutic window of the novel HDACi MPK77 in comparison to entinostat. Based on the IC_50_ obtained for the different HDACi (see Supplementary Table S1), we selected those HDACi for more detailed analysis regarding their genotoxic potential that showed an IC_50_ in the low micromolar range in malignant neuroblastoma cells, while being substantially (i.e., ≥10-fold) less cytotoxic to non-malignant V79 cells. These HDACi were considered as promising novel candidate HDACi because of their anticipated broader therapeutic window. Using these selection criteria, the hydroxamic acid-type HDACi KSK64, TOK77, and DDK137 as well as the benzamide-type HDACi MPK77 were chosen for further detailed investigations, aiming to identify HDACi with the lowest genotoxic hazard profile in V79 cells.

### 2.2. Formation of Micronuclei and DNA Strand Breaks by HDACi 

**Figure 2:**
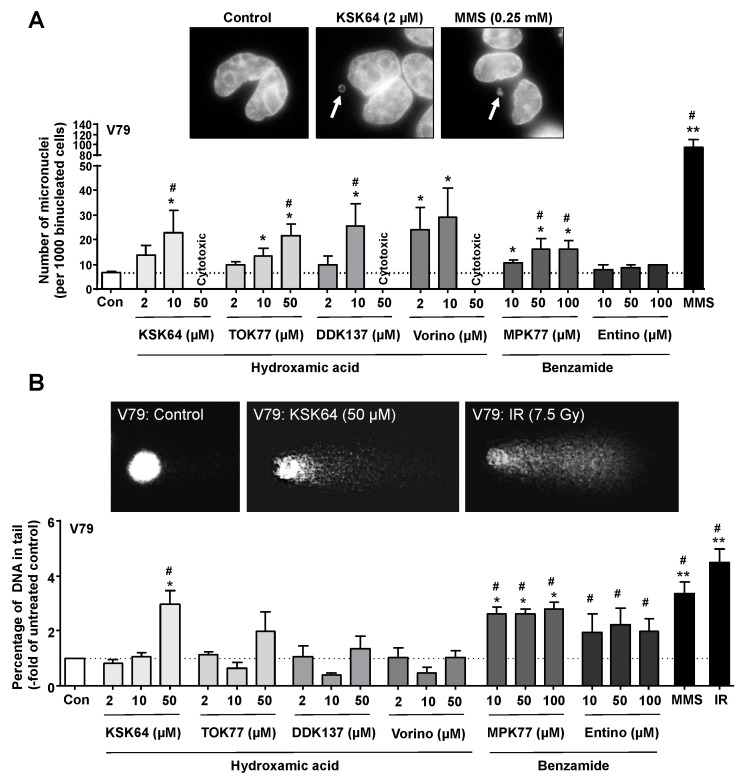
Genotoxic effects of hydroxamic acid- and benzamide-type HDACi as analyzed on the levels of micronuclei and DNA strand break formation. The formation of micronuclei (A) and DNA strand breaks (B) was analyzed after a 24-h treatment period of non-malignant V79 cells with representative HDACi candidate compounds. (**A**) The frequency of micronuclei was analyzed as described in Methods. Shown are representative pictures (the arrows point to micronuclei). Quantitative data (mean ± SEM) presented in the histogram were obtained from three independent experiments (n = 3) with each 1000 cells being analyzed per experiment; *, *p* ≤ 0.05; **, *p* ≤ 0.01 as compared to untreated control (Student’s *t*-test); # ≤0.1 (one-way ANOVA with Dunnett´s post hoc test). Data obtained from the use of 100 µM entinostat were obtained from a single experiment only (therefore, only the mean is shown and this data were excluded from statistical analyses). (**B**) DNA strand break formation was analyzed by the alkaline comet assay as described in methods. In the upper part, representative pictures are shown. Quantitative data presented in the histogram are the mean ± SEM (standard error of the mean) from three independent experiments (n = 3) with each 50 cells being analyzed per experiment; *, *p* ≤ 0.05; **, *p* ≤ 0.01 as compared to untreated control (Student´s *t*-test); # ≤0.05 (one-way ANOVA with Dunnett´s post hoc test). See also Supplementary Table S2 (% DNA in tail).

To investigate the potential of KSK64, TOK77, DDK137, and MPK77 to promote genetic instability, the micronucleus assay and the alkaline comet assay were employed. The alkaline comet assay detects DNA strand breaks (i.e., DNA single- (SSB) and DNA double-strand (DSB) breaks) as well as alkali-labile sites), while the micronucleus assay is indicative of chromosomal aberrations and, hence, can identify clastogenic and aneugenic substances. Vorinostat and entinostat were included as established reference drugs. Regarding chromosomal aberrations, we observed a significantly increased frequency of micronuclei following treatment of V79 cells with a concentration of ≥10 µM for each of the hydroxamic acid-type HDACi (Figure 2A). The benzamide-type HDACi MPK77 caused a slight but significant increase in the frequency of micronuclei, whereas entinostat did not (Figure 2A). These data show that all HDACi under investigation, except the benzamide-type HDACi entinostat, cause chromosomal aberrations in non-malignant V79 cells. The alkylating clastogen MMS (methyl methanesulfonate) was included for control and revealed substantial clastogenicity as anticipated (Figure 2A). 

Employing the alkaline comet assay, we found that the HDACi influenced the steady-state level of DNA strand breaks in V79 cells to different degrees (Figure 2B). A ≥2.0-fold and statistically significant increase in DNA strand break formation was observed with 50 µM of KSK64 (Figure 2B and Supplementary Table S2). Since cells floating in the supernatant were excluded from the comet analysis (see Methods), DNA fragmentation of apoptotic cells did not obscure the level of DNA damage observed in the comet assay. As opposed to KSK64, the HDACi TOK77, DDK137, and vorinostat did not cause a significant rise in DNA damage as detectable by the comet assay (Figure 2B). The benzamide-type HDACi MPK77 caused a significant increase in DNA strand breaks at all concentrations tested, similar to entinostat (Figure 2B). The alkylating agent MMS and ionizing radiation were used as positive controls and substantially increased the level of DNA strand breaks under our experimental setting as expected (Figure 2B). Overall, this set of data demonstrates that all HDACi under investigation reveal a genotoxic hazard as reflected either by the formation of micronuclei and/or DNA strand breaks. The observation that some compounds are genotoxic in the micronucleus assay but not in the comet assay is likely due to the different sensitivities of both assays to reflect DSB and SSB formation, respectively, or different mechanisms of DNA damage formation by the various HDACi. Among the hydroxamic acid-type HDACi, KSK64 showed the highest chromosomal aberration- (Figure 2A) and DNA strand break-inducing potency (Figure 2B) and, hence, is considered as the HDACi with the highest genotoxic potency. The novel HDACi TOK77 and DDK137 caused chromosomal aberrations (Figure 2A), while having the lowest capacity to induce DNA strand breaks as detected by the comet assay (Figure 2B). As compared to hydroxamic acid-type HDACi, the benzamide entinostat only promoted the formation of DNA strand breaks while MPK77 was also clastogenic.

### 2.3. Influence of HDACi on the Formation of Nuclear γH2AX (Ser139 Phosphorylated Histone H2AX) Foci

**Figure 3:**
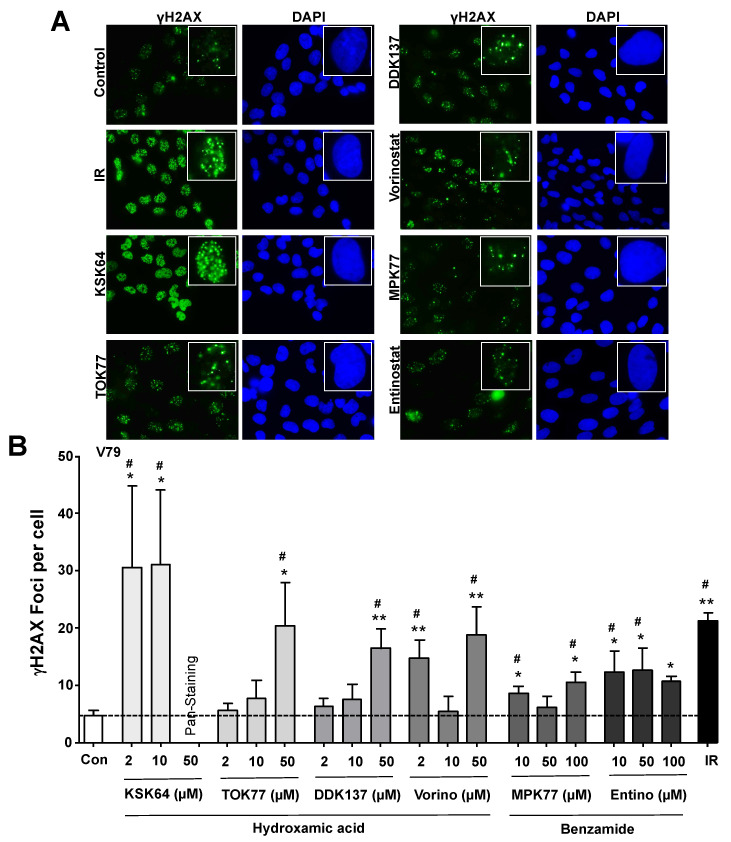
Hydroxamic acid- and benzamide-type HDACi stimulate the formation of nuclear γH2AX foci. The formation of nuclear γH2AX (Ser139 phosphorylated histone H2AX) foci was analyzed 24 h after the treatment of non-malignant V79 cells with representative HDACi candidate compounds. Ionizing radiation (IR) (radiation dose: 4 Gray (Gy)) was used as positive control and cells were analyzed 30 min after irradiation. **(A)** Representative pictures obtained following treatment with the hydroxamic acid-type HDACi KSK64, TOK77, DDK137, vorinostat (each 10 µM) or the benzamide-type HDACi MPK77 and entinostat (50 µM). **(B)** The number of nuclear γH2AX foci per cell is shown as the mean ± SEM from three independent experiments (n = 3). In each experiment 50 nuclei were evaluated; *, *p* ≤ 0.05; **, *p* ≤ 0.01 as compared to untreated control (Student´s *t*-test); # ≤ 0.05 (one-way ANOVA with Dunnett´s post hoc test).

In response to DNA damage induction, the so-called DNA damage response (DDR) becomes activated. The phosphoinositide 3-like (PI3-like) kinases Ataxia telangiectasia mutated (ATM) and ATM and Rad3-related (ATR), which are preferentially activated by DSB and stalled replication forks, respectively, are key players in the DDR, regulating cell cycle progression, DNA repair, and cell death [53,54,55]. Among others, the histone H2AX gets phosphorylated by ATM/ATR at position Ser139 (γH2AX). Therefore, the formation of nuclear γH2AX foci is considered as a sensitive surrogate marker of DNA damage, notably of DSBs [56]. We found a clear increase in the number of nuclear γH2AX foci following treatment of V79 cells with any of the hydroxamic acid-based HDACi, with KSK64 showing the most prominent effects already at a low concentration of 2 µM (Figure 3A,B). Since the IC_50_ of KSK64 for V79 cells is about 18 µM for a 24-h treatment period (Supplementary Table S1), the γH2AX foci data indicate that KSK64 can substantially impair genetic stability already at a very low cytotoxic concentration in non-malignant V79 cells. At a high concentration of 50 µM, nuclear γH2AX pan-staining was observed in the majority of KSK64-treated cells (Supplementary Figure S2 and Supplementary Table S3). The γH2AX pan-staining was originally demonstrated as a DSB-independent response to UV-induced DNA damage that results from nucleotide excision repair (NER)-induced DNA strand breaks [57]. In addition, γH2AX pan-staining can also be considered to reflect replicative stress. Overall, the γH2AX foci-based data support the hypothesis that all HDACi included in this study can cause DNA damage, including DSBs, in non-malignant V79 cells. It is also worth mentioning that the micronuclei data (see Figure 2) strengthen the view that the nuclear γH2AX foci observed upon HDACi treatment indeed reflect the formation of DSBs. Yet, it should be noted that TOK77 and DDK137 stimulate the formation of γH2AX foci only at a high dose of 50 µM, pointing to a relatively low DSB-inducing and DDR-activating potency of both hydroxamic acid-type HDACi as compared to KSK64 and vorinostat. Moreover, the relatively low number of γH2AX foci formed upon treatment of V79 cells with MPK77 and entinostat point to a reduced genotoxic hazard of these benzamide-type class I HDACi as compared to the hydroxamic acid-type pan HDACi. At first view, γH2AX foci data obtained with an intermediate concentration of vorinostat (i.e., 10 µM) may be assessed as a preliminary indication of a u-shaped dose-response curve of this HDACi. Yet, this speculation remains to be verified in forthcoming studies. 

**Figure 4:**
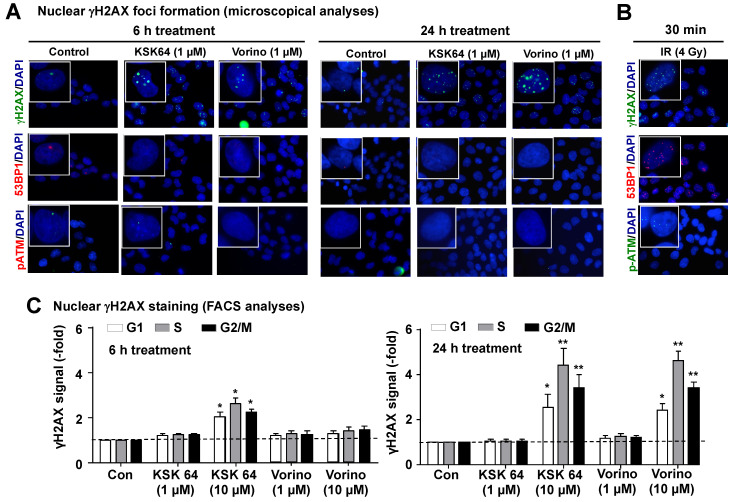
Cell cycle-related formation of DNA damage by hydroxamic acid- and benzamide-type HDACi. (**A**) Six to 24 h after addition of the hydroxamic acid-based HDACi KSK64 and vorinostat, the formation of γH2AX, 53BP1, and pATM foci (Ser1981 phosphorylated ATM) was analyzed in V79 cells. (**B**) For positive control, ionizing radiation (IR) was used. Shown are representative pictures of γH2AX, 53BP1, and pATM stained cells. (**C**) Six to 24 h after the addition of the hydroxamic acid-based HDACi KSK64 or vorinostat, the γH2AX signal intensity was determined in cells present in different phases of the cell cycle (i.e., in G1-, S-, and G2/M-phase) by flow cytometry-based method as described in Methods. Data shown are the mean ± SD from three independent experiments (n = 3); *, *p* ≤ 0.05; **, *p* ≤ 0.01 as compared to the untreated control, which was set to 1.0 (Student´s *t*-test).

Analyzing the time kinetics of γH2AX foci formation by KSK64 in more detail, we found a weak but clear increase in the number of nuclear γH2AX foci already at the early time point of 6 h after treatment of V79 cells with a very low concentration (i.e., 1 µM) of KSK64 (Figure 4A), which is in the range of the maximal blood serum concentration (C_max_) reported for vorinostat in humans [58]. The same holds true for the reference HDACi vorinostat (1 µM) as well as ionizing radiation (IR; 4 Gy), which was included as control (Figure 4A,B). Over time, the number of KSK64- and vorinostat-induced γH2AX foci further increased (Figure 4A). Notably, unlike IR, both KSK64 and vorinostat failed to cause the formation of nuclear 53BP1 foci (Figure 4A,B). Nuclear 53BP1 (p53 binding protein 1) foci are a further surrogate marker of DSBs, being indicative of a DSB repair pathway choice towards non-homologous end-joining (NHEJ), which rests on an interference of 53BP1 with BRCA1 function [59,60,61,62]. This finding indicates that KSK64- and vorinostat-induced DSB are not subject to repair by NHEJ. Lack of 53BP1 foci formation following HDACi treatment has also been observed by others and was related to an inhibition of BRCA1 and 53BP1 at the site of the DNA damage [24]. In addition, suppression of the expression of BRCA1, Chk1, and Rad51 recombinase was reported in acute myeloid leukemia (AML) cells following panobinostat treatment [31]. Of note, KSK64 and vorinostat also did not cause the formation of phospho-ATM (pATM) foci (Figure. 4A), whereas IR did so, as anticipated (Figure 4B). This finding indicates that the formation of nuclear γH2AX foci following treatment of non-malignant V79 cells with hydroxamic acid-type HDACi is independent of S1981-phosphorylated ATM. Recently, it was shown that HDACs contribute to the repair of interstrand cross-links (ICL) [26]. Interestingly, ICL can be generated by endogenously formed reactive aldehydes [63] and are well-known powerful inducers of replicative stress, which again is a major trigger of ATR-regulated stress responses [64]. Based on this literature data, we suggest that ATR-regulated mechanisms rather than ATM contribute to the formation of γH2AX foci in response to HDACi exposure. 

Considering the rapid appearance of nuclear γH2AX foci already 6 h after addition of low doses of KSK64, we speculated that KSK64-induced DNA damage formation may not be strictly related to a specific phase of the cell cycle. To address this point, we measured the γH2AX signal intensity as a function of time and HDACi concentration in cells that are present in the different phases of the cell cycle (i.e., G1-, S-, and G2/M-phase) by a flow cytometry-based method. Following a 6 h treatment period of V79 cells with KSK64, we found an about 2-fold increase in the γH2AX signal intensity in both G1-, S-, and G2-phase cells (Figure 4C). After a 24 h treatment period, the γH2AX signal intensity further increased (Figure 4C). Based on these data, we suggested that the hydroxamic acid-type pan HDACi KSK64 and vorinostat can cause DNA damage in all phases of the cell cycle. Collectively, we concluded that hydroxamic acid-type HDACi, including KSK64, trigger a rapid and cell cycle-independent formation of DNA damage, including DSBs, in non-malignant cells already at a low micromolar concentration and, furthermore, that the DSBs formed are presumably not subject to repair by NHEJ. 

### 2.4. Influence of HDACi on the Activation of Cell Death Pathways and Mechanisms of the DNA Damage Response (DDR) in Non-Malignant V79 Cells

**Figure 5:**
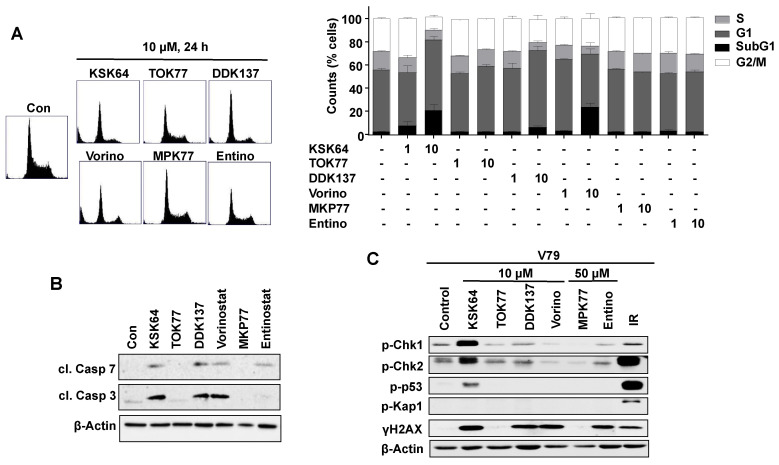
Influence of hydroxamic acid- and benzamide-type HDACi on the activation of cell death- and DDR-related mechanisms. (**A**) Cell cycle distribution was analyzed 24 h after the addition of hydroxamic acid- and benzamide-type HDACi to V79 cells, as described in Methods. In the left panel, representative data obtained from flow cytometry analysis are shown. On the right-hand side, the mean ± SD obtained from three independent experiments (n = 3) are shown. (**B**) Activation of caspase-mediated apoptotic death pathways was analyzed 24 h after treatment of V79 cells with 10 µM of the corresponding HDACi by Western blot analysis using antibodies that specifically detect the cleaved (i.e., activated) forms of caspase-7 (cl. Casp 7) and capase-3 (cl. Casp 3). The protein expression of beta-actin was used as protein-loading control. (**C**) The activation of selective factors of the DNA damage response (DDR) was analyzed by Western blot analysis of total cell extracts using phospho-specific antibodies as indicated. The protein expression of beta-actin was monitored for protein-loading control.

Measuring cell cycle distribution by flow cytometry, we did not observe an accumulation of V79 cells in the G2/M phase following HDACi treatment for 24 h (Figure 5A). Rather, a moderate increase of the percentage of V79 cells present in the SubG1 fraction, which is indicative of the apoptotic fraction, was observed following treatment with KSK64, vorinostat, and, to a lower extent, with DDK137 (Figure 5A). Cell death induced by KSK64, vorinostat, and DDK137 was accompanied by the activation of caspase-3 and -7 (Figure 5B), indicating that these HDACi trigger caspase-dependent apoptosis in non-malignant V79 cells. As opposed to the hydroxamic acid-based HDACi KSK64, DDK137 and vorinostat, the benzamide-type HDACi, entinostat, only activated caspase-7 but not caspase-3 (Figure 5B) and, furthermore, did not increase the percentage of cells present in the SubG1 fraction (Figure 5B). TOK77 and MKP77 neither induced cell death as reflected by an increase in the SubG1 population nor did they activate the executor caspases-3 and -7 (Figure 5A,B). This data point to remarkable variations of the various HDACi to trigger apoptotic cell death in non-malignant cells. Out of the different HDACi tested, TOK77 and MPK77 revealed the lowest pro-apoptotic activity in V79 cells, indicating that these two HDACi are characterized by a relatively low pro-apoptotic hazard.

Investigating the influence of the chemically different types of HDACi on mechanisms of the DDR by Western blot analyses, large HDACi-specific differences were again observed. KSK64 caused the most profound and widespread activation of DDR factors, as reflected by increased protein levels of γH2AX, p-p53, p-Chk1, and p-Chk2 but not of p-Kap1 (Figure 5C), which is in line with the result of the pATM foci analyses. DDK137, vorinostat, and entinostat also triggered a substantial increase in γH2AX levels but no, or only a minor, increase in p-p53 and p-Chk1/p-Chk2 levels, respectively (Figure 5C). Out of all the HDACi tested, TOK77 and MPK77 failed to provoke a clear increase in γH2AX protein level, supporting the view of a relatively low DDR-activating potency of these HDACi (Figure 5C). IR was used as a positive control and increases the levels of all phosphorylated DDR factors under investigation (i.e., p53, Chk1, Chk2, Kap1, H2AX) (Figure 5C). Since activation of checkpoint kinases is indicative of replicative stress, we speculate that especially the genotoxic effects observed for KSK64 are related to replicative stress and, accordingly, to ATR-regulated mechanisms. Hence, replication fork progression studies measuring BrdU (5-bromo-2´-deoxyuridine) incorporation are clearly required to scrutinize this hypothesis. We would like to mention that data obtained from the analyses of nuclear γH2AX foci are not always consistent with data obtained from the analysis of γH2AX protein levels by Western blot. This is because only nuclear γH2AX foci are specific and sensitive surrogate markers of DSBs while the γH2AX protein level detected in total cell extracts is indicative of different types of DNA damage [56,65,66,67]. Taken together, the data demonstrate that both hydroxamic acid-type pan-HDACi and class I-selective benzamide-type HDACi differently affect DDR mechanisms. Of note, the DDR profile of the novel hydroxamic acid-based HDACi KSK64 largely differs from that of the reference pan-HDACi vorinostat and the other HDACi under investigation (Figure 5C). This finding indicates that vorinostat and KSK64 either inhibit different classes of HDACs or inhibit an identical panel of HDACs, however, to a different extent. Summarizing, the data show that KSK64 is characterized by the most profound DDR-activating potency, while TOK77 and MPK77 reveal the weakest capacity to stimulate mechanisms of the DDR.

### 2.5. DSB Repair Defective Cells as a Tool to Predict the DSB-Inducing Potency of HDACi

**Figure 6:**
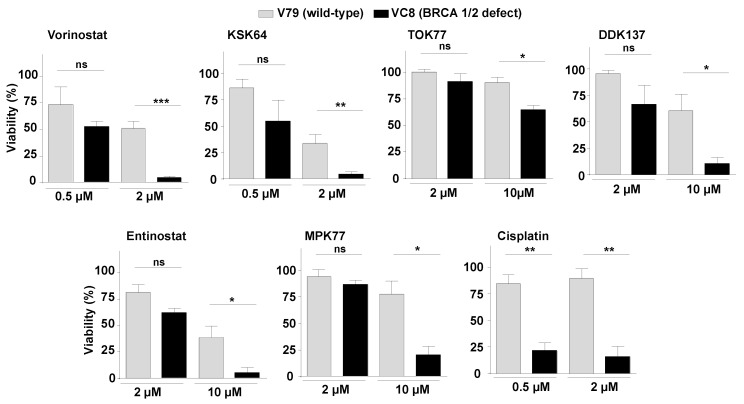
Sensitivity of DSB repair defective cells to hydroxamic acid- and benzamide-type HDACi. Viability of wild-type V79 cells and hamster mutant cells (VC8) that are deficient in DSB repair by homologous recombination (HR) due to a BRCA1/2 defect was analyzed 72 h after addition of HDACi by the Alamar blue assay, as described in Methods. The DNA intrastrand cross-linking agent cisplatin was used for control. Data shown are the mean ± SD from three independent experiments (n = 3) each performed in triplicate (N = 3); ns, not significant; *, *p* ≤ 0.05; **, *p* ≤ 0.01 as compared to the corresponding controls (Student´s *t*-test).

The results of the micronucleus assay, comet assay, nuclear γH2AX foci-based analyses, and DDR analyses consistently demonstrate that HDACi can cause DNA damage, including DSBs, in non-malignant V79 cells, although to differing extents. Keeping in mind that DSBs are a very potent trigger of cell death [68], we speculated that DSB repair-defective mutants are particularly sensitive to HDACi that can form DSBs. Thus, the viability of DSB repair-defective cells might be exploited as a predictive surrogate marker of the DSB-inducing potency of HDACi. Therefore, we investigated the effect of the various HDACi on the viability of VC8 cells, which are derived from V79 cells and are deficient in DSB repair by homologous recombination (HR) because of a BRCA1/2 defect [69,70]. Employing this cell model, we indeed found an increased sensitivity of VC8 cells to all of the HDACi under investigation, with TOK77 showing the weakest selective toxicity in the DSB repair defective hamster mutant (Figure 6). The data strongly support the hypothesis that both hydroxamic acid- and benzamide-type HDACi can promote the formation of cytotoxic DSBs, which are subject to DSB repair involving BRCA1/2-regulated HR. Moreover, these results are in line with the micronucleus- and γH2AX foci-based data arguing that chemically different types of HDACi can promote genetic instability. Interestingly, HR-defective VC8 cells are not only hypersensitive towards hydroxamic acid-type pan HDACi and benzamide-type class I HDACi (see Figure 6) but also the class I/IV selective benzamide-type inhibitor mocetinostat and the class I inhibitory depsipeptide romidepsin (Supplementary Figure S3). Assuming that DNA damage formation and genetic instability is an on-target effect of HDACi, this feature would be advantageous for its anti-tumor efficacy but it would be associated with an increased genotoxic hazard in normal cells. Such concern would also hold true for combination treatment regimen comprising HDACi and conventional anticancer therapeutics. 

**Figure 7:**
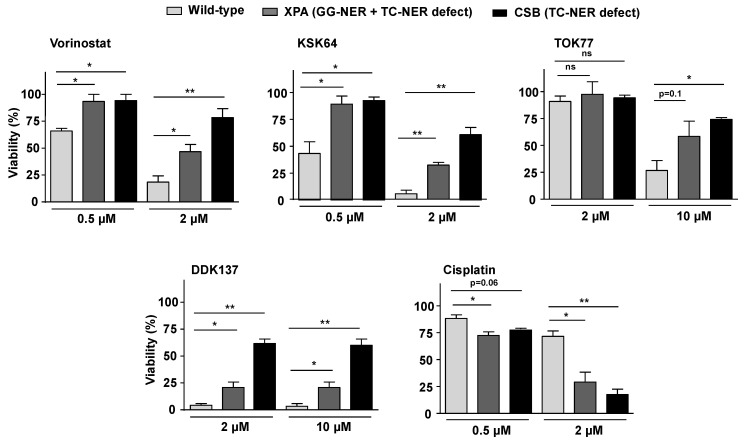
Sensitivity of nucleotide excision repair (NER) defective cells to hydroxamic acid- and benzamide-type HDACi. Viability of wild-type human fibroblasts and fibroblasts defective in nucleotide excision repair (NER) pathways (i.e., global genome nucleotide excision repair (GG-NER) or transcription-coupled nucleotide excision repair (TC-NER)) due to a defect in Xeroderma pigmentosum group A protein (XPA) or Cockayne syndrome protein B (CSB), respectively, was analyzed 72 h after the addition of HDACi by the Alamar blue assay, as described in Methods. The DNA intrastrand cross-linking agent cisplatin was used for control. Data shown are the mean ± SD from three independent experiments (n = 3) each performed in triplicate (N = 3); ns, not significant; *, *p* ≤ 0.05; **, *p* ≤ 0.01 as compared to the corresponding untreated controls (Student´s *t*-test).

The molecular mechanisms involved in the formation of DNA damage by HDACi are still unclear. Regarding hydroxamic acid-based pan-HDACi, it is speculated that they form bulky DNA adducts following Lossen rearrangement [40]. Yet, this speculation is mainly based on experiments using naked DNA. So, whether or not pan-HDACi can form bulky DNA adducts in a living cell is unclear. Since nucleotide excision repair (NER) is well known as the preferential DNA repair pathway for the repair of bulky DNA-lesions [71,72], we reasoned that NER defective cells should be hypersensitive to hydroxamic acid-type HDACi if these HDACi induce bulky DNA lesions. To test this hypothesis, we comparatively investigated the sensitivity of cells that are proficient or deficient in the NER scaffold protein Xeroderma pigmentosum group A (XPA) [73] to hydroxamic acid-type HDACi. XPA is essential for both global genome NER (GG-NER) and transcription-coupled NER (TC-NER) [71]. Unexpectedly, the data obtained show that XPA-deficient cells are even more resistant to all hydroxamic acid-type HDACi under investigation (i.e., vorinostat, KSK64, TOK77, DDK137) as compared to XPA proficient cells (Figure 7), with TOK77 revealing the weakest effect (Figure 7). Notably, XPA deficient cells are hypersensitive to the control compound cisplatin, as expected (Figure 7). Comparative analyses of cells lacking the Cockayne syndrome protein B (CSB), which is crucial for TC-NER and the repair of oxidative DNA damage [74,75], revealed identical results. CSB deficiency also resulted in an increased resistance to the hydroxamic acid-type HDACi, with TOK77 showing again the weakest effect (Figure 7). Based on the identical results obtained by the use of two different NER defective cell lines, we speculated that the Lossen rearrangement and subsequent formation of bulky DNA adducts does not majorly contribute to the genotoxic activity of hydroxamic acid-type pan-HDACi in intact cells. Forthcoming studies are required to clarify whether primary DNA adducts, other than bulky DNA adducts (e.g., DNA intra- or interstrand cross-links), are formed by HDACi, which are processed to highly cytotoxic secondary DNA lesions by help of the NER machinery.

A common feature of class I and II HDACi is that they are able to interfere with multiple pathways of DNA repair and DDR. Among others, some class I/II inhibitors have been shown to affect DSB repair of malignant cells by influencing BRCA1 interactions as well as the stability of DNA repair- and DDR-related proteins [24,25,28,76]. It is reasonable to assume that these mechanisms contribute to the anticancer efficacy of HDACi and, furthermore, that they constitute the molecular basis for additive or synergistic effects if they are used in combination with conventional anticancer drugs [30,31,36]. So, it appears that HDACi promote the formation of DSBs and increase genetic instability in malignant cells by impacting the bona fide processing of spontaneously generated DSBs that involves BRCA1/2-coordinated HR and Chk1-regulated DDR mechanisms. A highly important question is whether promotion of genetic instability as observed in the present in vitro study, especially for hydroxamic acid-type HDACi, is relevant for the in vivo situation. In this context, we would like to point out that belinostat has been demonstrated to cause genetic instability in an in vivo mouse model in human-relevant concentrations [51]. Whether the molecular mechanism(s) contributing to the genotoxicity of HDACi in non-malignant V79 cells are different from those in malignant cells remains to be elucidated in more detail in forthcoming studies, preferentially in vivo. To determine the molecular mode of genotoxic action of HDACi in normal cells and to define possible threshold concentrations for adverse effects is a highly important issue in view of the development of novel well-tolerated HDACi.

Taken together, both published in vitro and in vivo data, together with our results, imply that the promotion of genetic instability by currently available HDACi might be a class I/II-related effect resulting from on-target effects of these HDACi, which eventually influence DNA repair and DDR. Speculating that genetic instability results from a simultaneous inhibition of several HDACs, the identification of novel class- or isoform-specific HDACi may be a solution to the problem. In addition, having in mind that DNA repair may cause a practical threshold in chemical carcinogenesis [77,78], a patient´s individual genotoxic risk level may depend on his individual DNA repair and DDR capacity in normal cells as well as the dose of the respective HDACi. We suggest threshold concentrations of HDACi to be determined in preclinical in vitro and in vivo studies that would ensure a preferably low genotoxic risk profile of these compounds in terms of promoting genetic instability and activating the DDR in non-malignant cells. Hence, novel HDACi should undergo careful toxicological assessments to minimize their long-term genotoxic risk in personalized therapy, especially if used for the therapy of non-malignant diseases and childhood malignancies. Following this line of argument and comparing the genotoxic and cytotoxic/pro-apoptotic profile of the novel HDACi investigated in the study at hand (Table 1), KSK64 revealed the highest genotoxic and cytotoxic hazard and, hence, may be particularly useful as an anticancer agent to improve the therapy of highly malignant tumors. In contrast, TOK77 and MPK77 revealed the lowest genotoxic and cytotoxic hazard, which is indicative of their preferential tolerability and relatively weak chronic adverse effects and, hence, favor their application in non-malignant diseases. Clearly, forthcoming in vivo studies considering clinically relevant experimental settings are required to scrutinize these hypotheses. 

## 3. Materials and Methods 

### 3.1. Materials

For irradiation, a Cs-137 source (Gammacell 3000; Nordion, Ottawa, ON, Canada) was used. Vorinostat and entinostat were obtained from Sigma Aldrich GmbH (Taufkirchen, Germany). The compound library of hydroxamic acid- and benzamide-type HDACi derivatives was provided by T. Kurz and F. K. Hansen (Institute of Pharmaceutical and Medicinal Chemistry, Heinrich Heine University Duesseldorf, Düsseldorf, Germany). Synthesis and biological activities of the HDAC inhibitory compounds KSK64 [30] and DDK137 [79] were already described. More detailed information regarding the synthesis of the various HDACi is also provided by Pflieger et al. [80], Mackwitz et al. [81], and Krieger et al. [82]. Chemical structures of the HDACi tested are summarized in Supplementary Figure S1. HDAC inhibitory activity of the compounds was confirmed by analyses of hyperacetylation of histones as concluded from enzyme assays and cellular HDAC pan assays [20,30,33,79,82].

### 3.2. Chemical Synthesis of HDACi—Reaction, Monitoring, Purification, and Analytics

Chemical synthesis of HDACi has already been described in detail [30,79,80,81,82]. Chemicals and solvents were purchased from commercial suppliers (Sigma-Aldrich, (Taufkirchen, Germany); Acros Organics (Schwerte, Germany); TCI chemicals (Eschborn, Germany); Fluorochem ABCR (Karlsruhe, Germany); Alfa Aesar (Kandel, Germany); J&K chemicals (Altdorf, Germany); Carbolution (Saarbrücken, Germany)) and used without further purification. Dry solvents were obtained from Acros Organics. Ambient or room temperature corresponds to 22 °C. The reaction progression was monitored using Thin-Layer-Chromatography plates (ALUGRAM Xtra SIL G/UV254) from Macherey and Nagel (Düren, Germany). Visualization was achieved with ultraviolet irradiation (254 nm) or by staining with a KMnO4-solution (9 g KMnO4, 60 g K2CO3, 15 mL of a 5% aqueous NaOH-solution, adding 900 mL deionized water). Purification was either performed with prepacked Silica cartridges (RediSep^®^ Rf Normal Phases Silica, RediSep^®^ Rf RP C18) for flash column chromatography (CombiFlashRf200, TeleDyneIsco) or by recrystallization. Different eluent mixtures of solvents (hexane and ethyl acetate or dichloromethane and methanol) served as the mobile phase for flash column chromatography. An NMR-Spectrometer (Bruker Avance III-300, Bruker Avance DRX-500 or Bruker Avance III-600) by Brucker (Bremen, Germany) was used to perform ^1^H- and ^13^C-NMR experiments. Chemical shifts were given in parts per million (ppm), relative to residual non-deuterated solvent peak (^1^H-NMR: DMSO-d6 (2.50), 13C-NMR: DMSOd6 (39.52). Signal patterns were indicated as: Singlet (s), doublet (d), triplet (t), quartet (q), or multiplet (m). Coupling constants, J, were quoted to the nearest 0.1 Hz and were presented as observed. ESI-MS (electrospray ionization mass spectrometry) was carried out using Bruker Daltonics UHR-QTOF maXis 4G (Bruker Daltonics) under electrospray ionization (ESI). The above-mentioned characterizations were carried out by the HHU Center of Molecular and Structural Analytics at Heinrich-Heine University Düsseldorf (http://www.chemie.hhu.de/en/analytics-center-hhucemsa.html). APCI-MS (atmospheric pressure chemical ionization mass spectrometry) was carried out with an Advion expressionL compact mass spectrometer (CMS). Melting points were determined using a Büchi M-565 melting point apparatus (uncorrected). Analytical HPLC was carried out on a Knauer HPLC system comprised of an Azura P6.1L pump, an Optimas 800 autosampler, a Fast Scanning Spectro-Photometer K-2600, and a Knauer Reversed Phase column (serial number: FK36). Evaluated compounds were detected at 254 nm. The purity of all final compounds was 95% or higher.

### 3.3. Cell Culture and Treatment of Cells

V79 hamster lung cells, which are recommended in the OECD toxicology guidelines for hazard identification (i.e., cytotoxicity and genotoxicity) of chemicals, were purchased from the German Collection of Microorganisms and Cell Cultures (DSMZ, Braunschweig, Germany). They were cultured in Dulbecco´s modified Eagle medium and Ham´s F12 medium (DMEM/F12) (PromoCell, Heidelberg, Germany) containing 10% fetal calf serum at 37 °C in a humidified atmosphere containing 5% CO_2_. VC8 hamster cells were defective in DNA double-strand break (DSB) repair by homologous recombination (HR) due to the lack of functional BRCA2 protein [69,70]. VC8 cells were provided by B. Kaina (Institute of Toxicology, Mainz, Germany). Nucleotide excision repair defective XPA and CSB deficient cells were also provided by B. Kaina and were described before [83,84]. They were grown in DMEM containing 10% fetal calf serum. Human neuroblastoma cells (IMR-32 and SH-SY5Y) were purchased from the German Collection of Microorganisms and Cell Cultures (DSMZ, Braunschweig, Germany) and were used as a model of malignant cells. They were grown in DMEM or RPMI (Roswell park memorial institute) medium, respectively, containing 10% fetal calf serum. If not stated otherwise, HDACi were added to the exponentially growing cells and analyses were performed 6 h to 72 h later. 

### 3.4. Determination of Cell Viability and Cytotoxicity

Cell viability was determined using the Alamar blue assay [85], which reflected the mitochondrial activity. Viable cells were characterized by an effective mitochondrial metabolization of the non-fluorescent dye resazurin (Sigma, Steinheim, Germany) to fluorescent resorufin (excitation: 535 nm, emission: 590 nm). Relative viability in the untreated control was set to 100%. In addition, the Neutral red assay, which measures the integrity of lysosomal membranes, was used to monitor cytotoxicity. To this end, cells were incubated with the Neutral red solution (0.01%) in an atmosphere containing 5% CO_2_ for 90 min before they were fixed (1% formaldehyde/1% CaCl_2_). Afterwards, the Neutral red that was taken up by the cells was extracted (50% ethanol/1% of acetic acid, 15 min at room temperature (RT)) and absorbance (540 nm) was measured. If not stated otherwise, data are shown as the mean ± standard deviation (SD) of two to three independent experiments each performed in quadruplicate. Approximate IC_50_ (~IC_50_, see Supplementary Table S1) were calculated from the corresponding dose-response curves.

### 3.5. Cell Cycle Analysis

For flow cytometry-based analysis of cell cycle distribution, cells were trypsinized and combined with floating cells present in the medium. Following fixation with ice-cold ethanol (80%) (≥1 h, −20 °C), cells were pelleted (1000× *g*, 10 min, 4 °C) and suspended in phosphate-buffered saline (PBS). DNase-free RNase A (Serva, Heidelberg, Germany) was added (2 μg/mL, 1 h at RT). Nuclei were stained with propidium iodide (36.7 μg/mL Sigma, Steinheim, Germany) and analysis was performed using BD Accuri™C6 flow cytometer (BD, Franklin Lakes, NJ, USA).

### 3.6. Micronucleus Assay

This assay is frequently used in toxicological testing of clastogenic carcinogens and is part of the OECD test battery guideline suggested for genotoxicity testing of chemicals (http://www.oecd.org/env/ehs/testing/oecdguidelinesforthetestingofchemicals.htm). The assay scores the formation of chromosomal aberrations in interphase cells [86]. We analyzed the frequency of micronuclei in binucleated cells, as described [86]. Briefly, logarithmically growing cells were treated with cytochalasin B (3 µg/m) for 24 h. After fixation with formaldehyde (3.7% in PBS, 20 min, RT) and washing with PBS, cells were permeabilized using precooled methanol (20 min, −20 °C.) After washing with PBS and rinsing the cell layer with PBS containing Triton X100 (PBST; 0.3% Triton X100 in PBS), DNA was stained by DAPI (4´,6-diamino-2-phenylindole) and the number of micronuclei was evaluated by fluorescence microscopy. Data shown are the mean ± SEM of three independent experiments with ≥1000 binucleated cells evaluated per experimental condition. 

### 3.7. Alkaline Comet Assay

The formation of DNA strand breaks and apurinic/apyrimidinic sites was monitored employing the alkaline comet assay [87], which is part of the OECD guideline of in vivo genotoxicity testings and detects DNA strand breaks (DNA single- and double-strand breaks) and alkali-labile sites [87]. Briefly, after aspiration of the medium and trypsinization, cells were mixed with 0.5% melting agarose and transferred onto agarose-coated (1.5%) slides. After lysis in alkaline buffer (pH 10) (1 h, 4 °C, light protection) and unwinding of the DNA in precooled lysis buffer (pH >13) (20 min, 4 °C, light protection), electrophoresis was performed (electric current: 300 mA constant; 1 V/cm). After neutralization in salt buffer (pH 7.5) and staining with propidium iodide solution (50 µg/mL), comets were evaluated by fluorescence microscopy. Quantification of migrated DNA was performed with TriTek Comet Score™ software Version 1.5, evaluating ≥50 cells per condition (blinded assessment). Tail intensity (% DNA in tail) is displayed as the mean ± SEM of three independent experiments, each performed in duplicate (see Supplementary Table S2). Fold increase in tail intensities of treated cells were calculated by relating the % DNA in tail of treated cells to that of the corresponding untreated controls, which were set to 1.0 (see Figure 2B).

### 3.8. Analysis of DNA Double-Strand Break Formation 

The frequency of nuclear foci formed by S139 phosphorylated H2AX (γH2AX foci), which is a surrogate marker of DNA damage, especially DNA double-strand breaks (DSBs) [56,88], was assayed by immunocytochemistry-based analysis. As this assay reflects the genotoxic effects of chemicals [89,90] it complements the battery of genotoxicity tests suggested by the OECD guidelines for the testing of chemicals. The appearance of nuclear 53BP1 foci, which is another marker of DSBs [59,91], was also determined by immunocytochemistry. Cells were fixed with 4% formaldehyde in phosphate-buffered saline (PBS) (MERCK, Darmstadt, Germany) (15 min, RT) followed by incubation with ice-cold methanol (≥20 min, −20 °C). After blocking (1 h, RT, blocking solution: 5% BSA (MERCK, Darmstadt, Germany) in PBS/0.3% Triton X-100 (Sigma, Steinheim, Germany)), incubation with γH2AX antibody and 53BP1 antibody was performed (1:500, overnight, 4 °C). After incubation with the secondary fluorescence-labeled antibody (1:500, 1 h, RT, in the dark), cells were mounted in Vectashield (Vector Laboratories (Burlingame, CA, USA)) containing DAPI. The number of nuclear γH2AX and 53BP1 foci was scored (Olympus BX43 fluorescence microscope). Only nuclei with distinct foci were evaluated and γH2AX pan-stained nuclei were excluded from the foci analyses. Data are shown as the mean ± SEM of three independent experiments with ≥50 nuclei analyzed per experimental condition.

### 3.9. Western Blot Analysis

The activation of the DDR was investigated by Western blot analysis. To this end, total cell extracts were obtained by lysing an equal number of cells in Roti^®^-Load buffer (Carl Roth GmbH (Karlsruhe, Germany)) (5 min, RT). After sonication (EpiShear™ Probe sonicator, Active Motif, La Hulpe, Belgium), proteins were denatured by heating (5 min, 95 °C) and separated by SDS-PAGE (6% or 12.5% gel). Proteins were transferred onto a nitrocellulose membrane (GE Healthcare, Little Chalfont, UK) via the Protean Mini Cell System (BioRad (München, Germany)). The membrane was blocked with 5% non-fat milk in TBS/0.1% Tween 20 (MERCK, Darmstadt, Germany) (2 h, RT) and incubated with the corresponding primary antibody (1:1000, overnight, 4 °C). After washing with TBS/0.1% Tween 20, the secondary (peroxidase-conjugated) antibody was added (1:2000, 2 h, RT). The Fusion FX7 imaging system (PeqLab, Erlangen, Germany) was used for visualization of the bound antibodies. The following primary antibodies were used: Anti-Ser15 phosphorylated protein 53 (p-p53), anti-Talin-1, anti-Ser 345 checkpoint kinase 1 (p-Chk1), anti-cleaved caspase 3 and 7 (Cell Signaling, Beverly, MA, USA), anti-Ser824 phosphorylated Krüppel-associated box domain (KRAB)-associated protein-1 (p-Kap1), anti-Ser1981 phosphorylated ataxia telangiectasia mutated (pATM) and anti-Thr68 phosphorylated checkpoint kinase-2 (p-Chk2) (both Abcam, Cambridge, MA, USA), anti-Ser139 phosphorylated histone H2AX (γH2AX, Millipore, Billerica, MA, USA), and anti-β-actin (Santa Cruz, CA, USA). As secondary antibodies, horseradish peroxidase-conjugated secondary antibodies goat anti-mouse IgG and mouse anti-rabbit IgG (Rockland, Limerick, PA, USA) (2 h, RT) were used. 

### 3.10. Statistical Analyses

The Student´s *t*-test was used to demonstrate statistically significant differences between different HDACi-treated experimental groups and untreated controls. A *p* ≤ 0.05 was considered a statistically significant difference. In addition, one-way ANOVA with Dunnett´s post hoc test was performed.

## Figures and Tables

**Table 1:** Summary of the Genotoxic and Cytotoxic Potency of Novel Hydroxamic Acid- and Benzamide-Type HDACi.

HDACi	Comet Assay	Micronucl. Assay	gH2AX Foci	gH2AX Pan-Stain	DDR Activation	DSB Repair def. Cells	Apoptosis	Viability (IC_50_) 24 h - 72 h
*Vorinostat*	−	+	+	++	+	++	++	10 - 5
KSK64	++	+	++	+++	++	++	++	18 - 7
TOK77	−	+	+	0	−	+	−	>50 - >50
DDK137	−	+	+	+	+	+	+	33 - 11
*Entinostat*	−	-	+	+	+	+	0	>50 - 7
MPK77	+	+	+	0	−	+	−	>50 - 50

To monitor genotoxicity, we analyzed the impact of various types of HDACi on the formation of micronuclei and DNA strand breaks (i.e., alkaline comet assay and γH2AX foci assay) as well as γH2AX pan-staining and mechanisms of the DDR using non-malignant V79 lung hamster fibroblasts. Moreover, the influence of the DSB repair status on the cellular sensitivity to HDACi was investigated by comparative analysis of DSB repair proficient (V79) versus DSB repair deficient (VC8) mutants. In addition, induction of apoptotic cell death was calculated on the basis of the caspase-3,-7 cleavage and SubG1 population. Cell viability was determined 24 h and 72 h after drug treatment and presented as half maximal inhibitory concentration (IC_50_) (depicted in µM). Original data are shown in the corresponding figures. The hydroxamic acid-type pan-HDACi vorinostat and the benzamide-type class I HDACi entinostat (written in italic) were used as reference compounds; −, no effect; 0, weak effect; +, moderate effect; ++, strong effect; +++ very strong effect as compared to untreated controls.

## Supplementary Materials

Supplementary Materials can be found at https://www.mdpi.com/1422-0067/21/13/4747/s1. Figure S1: Chemical structures of HDACi used in the present study. Figure S2: Analysis of nuclear γH2AX pan-staining induced by various HDACi. Figure S3: Viability analysis of wild-type and DNA repair defective hamster cells to the HDACi mocetinostat and romidepsin. Table S1: Comparative characterization of the cytotoxic activity of various HDACi in non-malignant V79 cells versus two human neuroblastoma cell lines. Table S2: HDACi-induced increase in the “percent DNA in tail” as analyzed by the Comet assay. Table S3: Genotoxic potency of HDACi in V79 cells as analyzed on the levels of nuclear γH2AX foci formation and nuclear γH2AX pan-staining.

## Abbreviations

ATM	Ataxia telangiectasia mutated
ATR	ATM- and Rad-3 related
BRCA1/2	Breast cancer associated gene 1/2
Chk1/2	Checkpoint kinase 1/2 (CHEK1/2)
CSB	Cockayne syndrome protein B
DDR	DNA damage response
DSB	DNA double-strand breaks
ERK2	Extracellular regulated kinase 2
γH2AX	Ser139 phosphorylated histone H2AX
HDACi	Histone deacetylase inhibitor
HR	Homologous recombination
Kap1	KRAB-associated protein 1 (TRIM28)
NER	Nucleotide excision repair
NHEJ	Non-homologous end-joining
XPA	Xeroderma pigmentosum complementation group A
