# Supplementary material for: In Vitro Assessment of the Genotoxic Hazard of Novel Hydroxamic Acid- and Benzamide-Type Histone Deacetylase Inhibitors (HDACi)
Source: Int J Mol Sci. 2020 Jul 3;21(13):4747. doi: 10.3390/ijms21134747 (PMC7370100; doi:10.3390/ijms21134747)
Supplement: Supplementary file 1 [file ijms-21-04747-s001.pdf]

HDACi	Chemical structure	Type of HDACi
MPK264	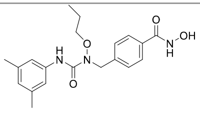	Hydroxamic acid
LAK41	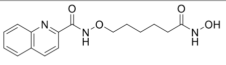	Hydroxamic acid
KSK64	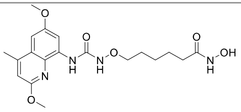	Hydroxamic acid
LAK39	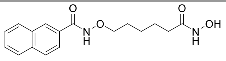	Hydroxamic acid
ABK-86	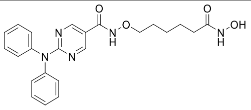	Hydroxamic acid
TOK77	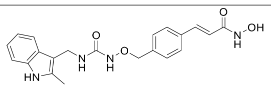	Hydroxamic acid
DDK137	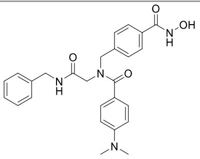	Hydroxamic acid
VTK36	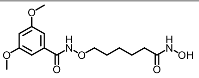	Hydroxamic acid
Vorinostat	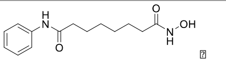	Hydroxamic acid
LAK88	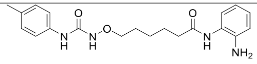	Benzamide
MPK211	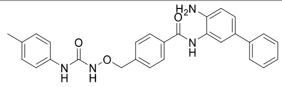	Benzamide
MPK77	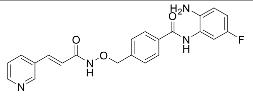	Benzamide
VSKKKK1-NH_2	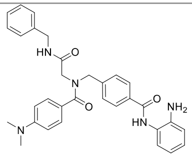	Benzamide
Entinostat	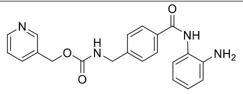	Benzamide

Supplementary Figure A1: Chemical structures of hydroxamic acid- and benzamide-type HDACi tested in the present study. Vorinostat and entinostat, which are well-established and clinically used HDACi, were included for control. Following initial cytotoxicity analyses of this HDACi compound library, the HDACi KSK64, TOK77, DDK137, and MPK77 revealed the most preferential therapeutic window and, hence, were pre-selected for further genotoxicity testing.

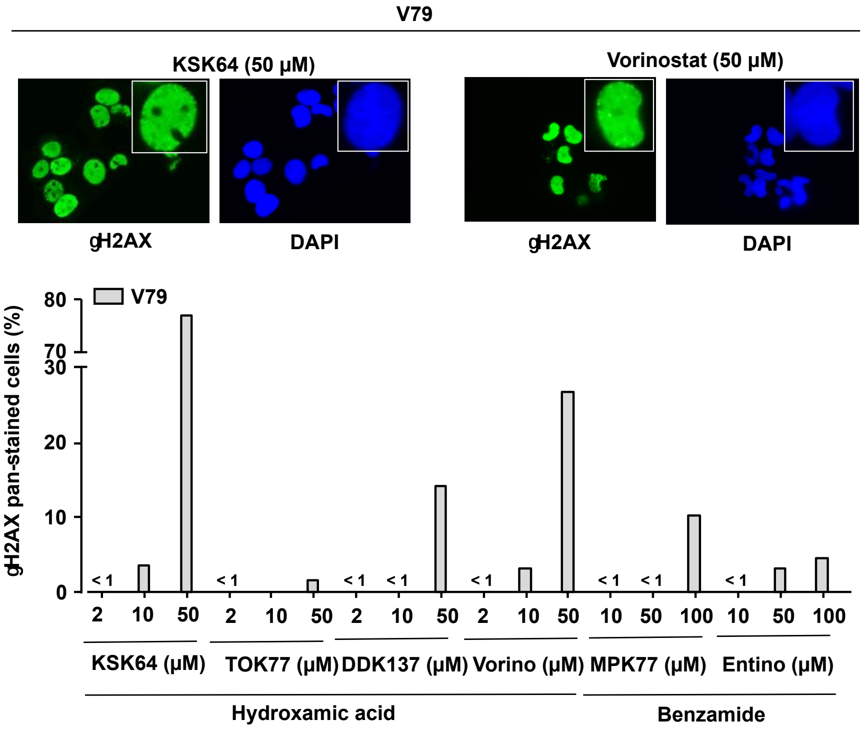

Supplementary Figure A2: High dose of hydroxamic acid- and benzamide-type HDACi stimulate γH2AX pan-staining. The frequency of γH2AX pan-stained nuclei was analyzed 24 h after treatment of non-malignant V79 cells with representative HDACi candidate compounds. Upper panel: Representative pictures; lower panel: The percentage of pan-stained cells is shown as mean value from three independent experiments (n = 3); <1; less than 1% of cells showed γH2AX pan-staining. In each experiment 50 nuclei were evaluated. See also Supplementary Table 2.

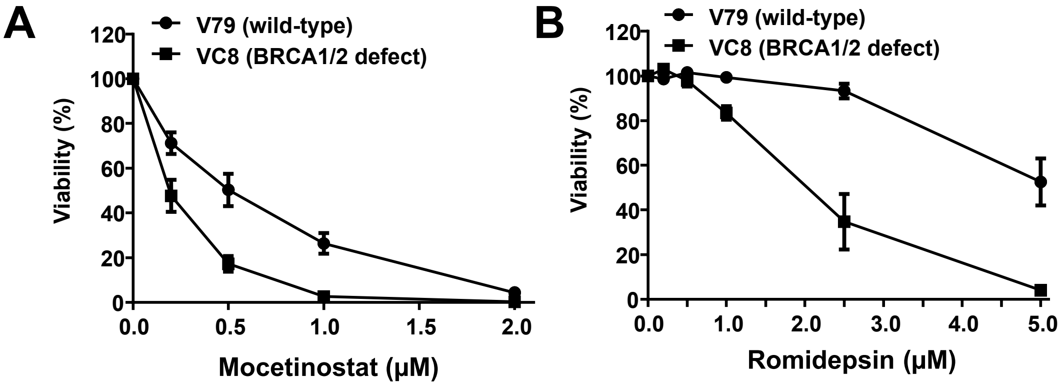

Supplementary Figure A3: Sensitivity of DNA repair defective cells to the HDACi mocetinostat and romidepsin. Viability of wild-type cells (V79) and cells defective in DSB repair by homologous recombination (VC8) was analyzed 72 h after the addition of the HDACi mocetinostat (A) or romidepsin (B) by the Alamar blue assay, as described in Methods. Data shown are the mean ± SD from at least three independent experiments (n = 3), each performed in triplicate (N = 3).

Supplementary Table B1: Comparative Characterization of the Cytotoxic Potency of Hydroxamic Acid- and Benzamide-Type HDACi.

		HDACi	IC_{50} - 24 h Treatment			IC_{50} - 72 h Treatment		
			V79	SH-SY5Y	IMR-32	V79	SH-SY5Y	IMR-32
Alamar Blue Assay	Hydroxamic Acid	MPK264	~ 22	~ 10	~ 12	~ 19	~ 3	~ 2
		LAK41	~ 10	~ 1	~ 2	~ 4	~ 0.2	~ 0.2
		KSK64	~ 18	~ 6	~ 0,8	~ 7	~ 0.5	~ 0.3
		LAK39	~ 10	~ 3	~ 4	~ 7	~ 0.3	~ 0.1
		ABK-86	> 50	> 50	> 50	> 50	~ 18	~ 9
		TOK77	> 50	> 50	~ 8	> 50	~12	~ 3
		DDK137	~ 33	~ 50	~ 0.6	~ 11	~ 0.9	~ 0.2
		VTK36	~ 7	~ 10	~ 0.9	~ 27	~ 2	~ 0.3
		Vorinostat	~ 10	~ 33	~ 5	~ 5	~ 1	~ 0.6
	Benzamide	LAK88	> 50	~ 15	~ 15	> 50	~ 4	~ 10
		MPK211	> 50	> 50	> 50	~ 25	~ 9	~ 15
		MPK77	> 50	~ 33	~ 3	~ 50	~ 17	~2
		VSKKKKK1-NH2	> 50	~ 47	~ 10	~ 4	~ 5	~ 0.9
		Entinostat	> 50	> 50	~50	~ 7	~ 8	~ 2
Neutral Red Assay	Hydroxamic Acid	MPK264	~ 20	~ 24	~ 16	~ 17	~ 3	~ 2
		LAK41	~ 9	> 50	~ 10	~ 3	~ 0.4	~ 0.3
		KSK64	~ 19	~ 12	~ 0.8	~ 6	~ 0.4	~ 0.3
		LAK39	~ 26	> 50	~ 0.7	~ 6	~ 0.6	~ 0.3
		ABK-86	> 50	> 50	~ 45	> 50	~ 26	~ 17
		TOK77	> 50	> 50	~ 50	> 50	~ 17	~ 5
		DDK137	~ 36	> 50	~ 10	~ 17	~ 1	~ 0.3
		VTK36	~ 21	~ 19	~ 10	~ 7	~ 5	~ 0.3
		Vorinostat	~ 8	~ 50	> 50	~ 1	~ 2	~ 0.6
	Benzamide	LAK88	> 50	> 50	~ 25	> 50	~ 2	~ 11
		MPK211	~ 32	> 50	~ 50	~ 25	~ 15	~ 15
		MPK77	> 50	> 50	~ 9	~ 35	~ 23	~ 3
		VSKKKKK1-NH2	> 50	~ 31	~ 28	~ 4	~ 6	~ 1
		Entinostat	> 50	> 50	> 50	~ 2	~ 9	~ 3

To monitor cell viability, both the Alamar blue and the Neutral red assay were applied to calculate IC_{50} values (μM concentrations shown) following 24 h or 72 h of treatment. As model for malignant cells, the neuroblastoma cell lines IMR-32 and SY-SY5Y were used. As a non-malignant cell model, V79 lung hamster fibroblasts were used, because this model is part of pre-clinical cyto- and genotoxicity testings, according to international OECD guidelines. Data shown are the mean from n ≥ 2 independent experiments each performed in quadruplicate (N = 4). Approximate IC_{50} values were calculated from the graphs of the corresponding dose response curves.

Supplementary Table B2: Genotoxic Effects of Hydroxamic Acid- and Benzamide-Type HDACi, as Analyzed on the Level of DNA Strand-Break Formation.

	**Mean (% DNA in tail)**	**± SEM**
Control (untreated)	16.8	1.9
KSK64 (2 µM)	12.9	6.5
KSK64 (10 µM)	19.5	1.3
KSK64 (50 µM)	37.8 #	6.1
TOK77 (2 µM)	16.7	5.7
TOK77 (10 µM)	11.1	2.7
TOK77 (50 µM)	25.2	9.3
DDK137 (2 µM)	15.1	5.3
DDK137 (10 µM)	6.9 #	0.2
DDK137 (50 µM)	17.3	5.6
Vorinostat (2 µM)	14.0	4.9
Vorinostat (10 µM)	8.0	2.8
Vorinostat (50 µM)	13.2	2.9
MPK77 (10 µM)	34.0 #	5.0
MPK77 (50 µM)	48.1 #	4.2
MPK77 (100 µM)	35.5 #	3.5
Entinostat (10 µM)	34.5 #	6.9
Entinostat (50 µM)	37.8 #	1.9
Entinostat (100 µM)	34.9 #	2.5
MMS (0.25 mM)	50.9 #	6.3
IR (7.5 Gy)	64.9 #	4.6

DNA strand-break formation was analyzed after a 24-h treatment period of non-malignant V79 cells with representative HDACi candidate compounds using the alkaline comet assay, as described in Methods. Quantitative data (% DNA in tail) shown are the mean ± SEM from three independent experiments with each 50 cells being analyzed per experimental condition; # $p \leq 0.05$ (one-way ANOVA with Dunnett´s post hoc test).

Supplementary Table B3: Comparative characterization of the genotoxic hazard of hydroxamic acid- and benzamide-type HDACi.

V79	Dose	≤ 5 Foci [%]	> 5 < 30 Foci [%]	≥ 30 Foci [%]	Pan-Stained [%]	Total [%]
Control	0 µM	72.9	26.9	0.,3	0.0	100
	2 µM	37.1	34.6	28.0	0.3	100
KSK64	10 µM	28.9	22.1	45.4	3.6	100
	50 µM	0.0	12.0	10.9	77.1	100
	2 µM	66.1	34.0	0.0	0.0	100
TOK77	10 µM	61.4	37.3	1.2	0.0	100
	50 µM	35.5	44.9	17.9	1.7	100
	2 µM	60.8	38.5	0.7	0.0	100
DDK137	10 µM	56.4	40.1	3.5	0.0	100
	50 µM	31.3	36.8	17.8	14.1	100
	2 µM	39.3	54.7	6.0	0.0	100
Vorinostat	10 µM	75.9	18.5	2.4	3.2	100
	50 µM	23.6	34.2	15.6	26.7	100
	10 µM	49.6	49.9	0.6	0.0	100
MPK77	50 µM	58.6	41.5	0.0	0.0	100
	100 µM	35.5	47.9	6.5	10.2	100
	10 µM	45.2	43.6	10.8	0.4	100
Entinostat	50 µM	45.3	39.4	12.2	3.2	100
	100 µM	41.7	47.2	6.5	4.6	100
Irradiation	4 Gy	2.3	76.3	21.4	0.0	100

The formation of nuclear γH2AX foci was analyzed 24 h after treatment of non-malignant V79 cells with representative HDACi candidate compounds. The percentage of cells showing γH2AX foci formation was calculated and cells were sub-grouped into four categories: Cells with low foci number (≤5 foci/cells); medium foci number (5–30 foci/cells); high foci number (≥30 foci); cells showing γH2AX pan-staining (pan-stained cells). Ionizing radiation was used as positive control. Data shown are mean values obtained from at least three independent experiments (n ≥ 3).
